# Tracking Listening Skill Development in Infants and Children with Hearing Loss: A Normative Dataset for the Functional Listening Index—Paediatric (FLI-P^®^)

**DOI:** 10.3390/children11091052

**Published:** 2024-08-28

**Authors:** Robert S. C. Cowan, Aleisha Davis, Pia Watkins, Katie Neal, Ruth Brookman, Mark Seeto, Janette Oliver

**Affiliations:** 1 Department of Audiology and Speech Pathology, The University of Melbourne, Melbourne, VIC 3053, Australia; r.cowan@unimelb.edu.au; 2 The Shepherd Centre, Sydney, NSW 2042, Australia; pia.watkins@shepherdcentre.org.au (P.W.); katie.neal@shepherdcentre.org.au (K.N.); 3 MARCS Institute, Western Sydney University, Penrith, NSW 2751, Australia; ruth.brookman@westernsydney.edu.au; 4 National Acoustic Laboratories, Sydney, NSW 2109, Australia; 5 Cochlear Limited, Sydney, NSW 2113, Australia

**Keywords:** FLI-P, functional listening, early intervention, hearing loss, paediatric, normative data, tracking progress

## Abstract

Background: Longitudinal studies highlight the importance of early intervention and timely device fitting for language development in children with congenital or early acquired hearing loss. Due to the variability in hearing loss, comorbidities, family circumstances, and service access, individualised monitoring of listening development is essential to inform decision-making. The Functional Listening Index—Paediatric (FLI-P), a 64-item hierarchical checklist of listening skills, has been validated for children with hearing loss aged 0–6 years. This study aimed to develop benchmarks for the FLI-P in typically hearing children, allowing for comparison with individual children with hearing loss. Methods: FLI-P scores were obtained from parents/caregivers of 561 typically hearing children aged 0–72 months. Each child’s FLI-P score was categorised into a 6-month age block, with a minimum of 36 data points per block. Quantile regression was employed to establish percentiles of FLI-P scores by age. Results: FLI-P scores were successfully recorded for all 561 children. Regression analysis determined that the 16th and 84th percentiles of FLI-P scores corresponded to approximately ±1 standard deviation from the median score for each age group. A graphical representation of these percentile trajectories was created to facilitate comparison between children with hearing loss and the normative data. Conclusion: A normative dataset of FLI-P scores from typically hearing children has been established, allowing for comparisons with the scores and developmental trajectories of individual children with hearing loss. The study demonstrates how FLI-P can guide early intervention decisions and effectively monitor progress.

## 1. Introduction

Over the past decade, research focused on child development has established the sensitivity of the early years of brain development in children and that physiological, pathological, socio-environmental, and familial factors impact the trajectory of that development [1,2,3]. It is well established that the tools for monitoring early childhood development and the comparative data enabling the monitoring of progress (relative to age-appropriate benchmarks, particularly in children who have developmental risks [4,5]) can provide valuable information to identify if and where gaps in development exist and guide decisions for appropriate supports for both professionals and parents [6,7,8].

The importance of hearing in the development of language and communication in infants and young children has been well-documented [9,10,11]. Audition and listening difficulties can result in speech and language development delays, as well as subsequent lower academic and educational achievement [12,13], decreased employment opportunities [14], and impacts on social cognitive development [15], mental health, and quality of life [16,17,18]. Early language skill development investigations in children with hearing loss have historically relied on a collection of outcome data at regular intervals as the child ages, typically at 6- or 12-month intervals [19,20]. Reliable measurements of language development using age-appropriate assessments can be particularly difficult for infants and young children with hearing loss [21,22]. Assessments conducted at long intervals may miss critical development periods, as significant milestones may be achieved more frequently than every 6–12 months [23,24]. Importantly, regular and continuous monitoring is crucial to promptly identify and address developmental changes or delays [24]. Children with hearing loss may show particular variable developmental trajectories, requiring more frequent evaluations to accurately track their progress [25,26] and promptly ensure modifications and targeting of clinical management.

Nott et al. [27] reported on the increased interest in developing parent-reporting techniques to address the need for more frequent assessments of language development in young children with hearing loss, as well as the benefits of the direct involvement of parents in tracking language acquisition. Graphical information can significantly enhance these techniques by providing clear and interpretable visualisation of data [28], which can assist and support the family in the process of self-advocacy [29], increase parental engagement and empowerment [30,31], and improve speech and language outcomes [32,33].

The development of checklists for spoken language acquisition and speech production that rely on parent-reporting techniques has stressed the importance of parallel development of norms for their use for children with typical hearing [34,35]. Normative data sets for specific checklists/tools obtained from linguistic and cultural age-appropriate normative populations have proven beneficial in speech pathology by providing benchmarks for typical development and identification of deviations from those norms [35,36,37]. Clinicians use these data sets to assess primary care decision outcomes and manage individuals with specific disorders [7,38]. Moreover, normative data are integral to routine clinical practice, aiding in the identification of delays, monitoring progress, and tailoring interventions based on individually assessed progress compared to age-appropriate norms [36,39,40].

Additionally, monitoring tools for child development with graphical interfaces provide real-time feedback, aiding in the proactive and effective management of developmental concerns [41,42] and supporting parents and clinicians to identify areas where a child may need additional support more promptly [40].

As the development of listening skills is a necessary precursor to the acquisition of spoken language [24,43,44,45,46], normative listening data can provide early evidence for clinicians and parents to support early decisions, even before delays in speech and language become apparent. Evidence that enables changes in clinical management and intervention can lead to improved speech and language outcomes, as well as digestible, accessible information for parents on how their child is progressing against peer-equivalent norms.

The Functional Listening Index—Paediatric has been developed to track the acquisition of a child’s listening skills over time and provide a graphic trajectory of developing listening competency. The feasibility and validity of the FLI-P have been reported previously [47], and the FLI-P has been used to clinically evaluate auditory skill development in children with congenital hearing loss [47,48]. Davis et al. [47] evaluated the FLI-P in 543 children with hearing loss enrolled in an early intervention and cochlear implant program over 6 years. The results established that the FLI-P was a viable clinical measure that could be used to monitor the development of listening skills in a wide range of children of different ages, degrees of hearing loss, device use, and additional support needs. Children’s individual FLI-P scores against age and aggregated group data were consistent with expected differences and variations over time and were sensitive to factors known to impact listening.

In addition to the children with hearing loss, Davis et al. [47] also assessed FLI-P scores in a small group of 32 typically hearing children, all of whom had been assessed as having language at age-appropriate levels. FLI-P results for this typically hearing group indicated a linear relationship over time, and comparison with the group of children with hearing loss indicated expected differences (the typically hearing children showed less variation and more developed listening skills across age ranges). However, given the relatively small number of children in the typically hearing group, it was not possible to develop trajectories for the development of listening skills for children from birth to 6 years of age that were robust and could be used for comparative evaluation of progress in auditory development.

The primary aim of this study was to establish a robust normative dataset on the listening skills of typically hearing children from birth to 6 years (0–72 months) for use as benchmarks and to enable comparison of the progress of children with hearing loss against those norms. A secondary objective was to examine how normative listening data facilitate tracking of individual progress to inform clinical recommendations and decision making.

## 2. Materials and Methods

### 2.1. Study Design

This study employed a multi-center cross-sectional observational design to collect data from a large population of children in Australia, aged birth to 6 years. Parents or caregivers who had previously attended one of three clinical research programs (MARCS Institute Baby Lab, Macquarie University Child Language Lab, or The Shepherd Centre) with their children were invited to participate via email, in-person during their visit, or online through a link. Participation was entirely voluntary, and parents could withdraw at any time. Data were collected during a single visit, either in person or via an electronic link sent to the identified parents/caregivers via email. Data were digitally stored after collection and were anonymised to remove personal identification details.

Data collection procedures were designed to enable parents to complete the online version of the FLI-P, which incorporated instructions for self-completion. Additionally, instruction from an investigator trained in using the FLI-P or access to a paper-based format was accessible if required by a parent.

For those children meeting inclusion/exclusion criteria, in addition to the FLI-P data, demographic information was also collected.

Parents/caregivers were asked to record observations of the FLI-P questions for their child. Parents were advised that the purpose of the FLI-P was to provide a “snapshot” of their child’s responses to sound, listening abilities, and how their child used information that they heard in daily life within the family and their community/environment.

In providing answers, parents/caregivers were provided with written instructions that helped them determine their responses; for example, they were asked to remember the following responses:

- “Mostly” means the child does it regularly, in different places, with different people;
- “Rarely” means that the child has only done it occasionally, a few times, or not at all.

The FLI-P includes specific questions related to the following:

- Listening, e.g., Q1: Jumps or startles to loud noises;
- Associating sound with meaning, e.g., Q8: makes sounds back to me when I talk to them;
- Comprehending simple spoken language, e.g., Q19: repeats three familiar sounds after me;
- Comprehending simple language in different listening conditions, e.g., Q31: follows short directions that are unpredictable or silly;
- Listening through discourse and narrative, e.g., Q42: recognises a familiar person on the phone;
- Advanced listening skills, e.g., Q56: can have a simple conversation with a familiar person on the phone.

### 2.2. Test Material: The Functional Listening Index—Paediatric

The Functional Listening Index—Paediatric (FLI-P) has been described in detail previously [47].

The FLI-P comprises a checklist of 64 specific statements (as noted in the examples above) that can be either administered on paper or in a digital format. For this study, it was developed into an internet-based application that could be accessed by health professionals or parents/caregivers using their personal computer, tablet, or an online device. Responses in auditory development questionnaires are often arranged in a sequential order reflecting the typical progression of auditory skills, as outlined by experts like Ling, Pollack, and Estabrooks. According to Ling [49], auditory skills develop predictably, starting with detection, moving through discrimination and identification, and culminating in comprehension. Pollack [50] emphasised that questionnaires should mirror this sequential learning to accurately assess a child’s auditory capabilities at each stage. Estabrooks [51] further supports the need for a structured hierarchy in auditory skill development assessment, advocating for a range of items that span different levels of ability.

To address these challenges, responses in the FLI-P are designed to reflect stages of skill acquisition. Specific abilities are organised across six hierarchical areas: sound awareness, associating sound with meaning, comprehending simple spoken language, comprehending language in different listening conditions, listening through discourse and narratives, and advanced open-set listening (see Appendix A). For each statement (e.g., jumps or startles to loud sound), the parent or professional assesses their observation of the child as related to the statement. Rather than a “yes/no” response, the rater is required to select either a “mostly” or “rarely” response to determine if a child has largely acquired a skill (i.e., mostly: demonstrates it regularly, in different places, with different people) or has not yet developed a listening skill (i.e., rarely: has demonstrated it occasionally, a few times, or not at all).

Early childhood developmental and milestone checklists, such as the FLI-P, typically assess the presence or absence of skill, behaviour, or knowledge with yes/no questions [52]. However, skill acquisition is not an “all or nothing” concept; there are levels of proficiency [53,54]. This makes categorising achievement in a yes/no format challenging, as children may use a skill inconsistently as they develop. Competency checklists address this issue by using scales of proficiency like “beginning”, “developing”, “competent”, or “mostly at this age” and “roughly around this time” [55,56].

Feedback from the FLI-P indicated that recording the date of “checked off” items was problematic, as it did not reflect the true date the child acquired the skill. A review of checklists and scales used in communication measures and competency development in health, education, and disability fields suggested that a two-item rating scale (“mostly”, “rarely”) would be more appropriate. This approach, as used in the Social Attention and Communication Surveillance System (SACS-R) [57], helps address these challenges. “Mostly” indicates that the child consistently and easily demonstrates the skill in various contexts, while “rarely” indicates the skill is shown infrequently or with difficulty.

For each administration of the FLI-P, a basal and a ceiling score is assessed. The basal is assessed by evaluating the four latest items with “mostly” responses to check for changes. A ceiling is reached once a sequence of responses to test items has been recorded as “rarely”. Once a ceiling is reached, the overall score for that point in time is calculated as the total number of items for which “mostly” has been recorded for that individual child, and this score is then plotted against the child’s age in months at the time of the assessment. This then enables a trajectory of FLI-P score against age to be drawn for an individual child, which can be used by the parent or professional in assessing progress for that child and in comparison to children with similar characteristics of hearing loss or group results for typically hearing children at similar age points.

#### 2.2.1. Subjects

Subjects were drawn from three separate institutions, the MARCS Baby Lab at Western Sydney University, The Shepherd Centre, and the Macquarie University Child Language Lab.

Inclusion criteria for the study included:

1. Children aged between birth and 6 years of age (0–72 months);
2. Pass on Newborn Hearing Screen Status conducted during the New South Wales Statewide Infant Screening—Hearing (SWISH) Program;
3. No current parental or professional concerns regarding hearing status.

Children were excluded from the study if any of the following criteria were present:

1. Pre-existing diagnosis of additional needs;
2. Unrealistic expectations on the part of the subject or the subject’s parents or carers regarding the possible benefits, risks, and limitations of the study;
3. Unwillingness or inability of the subject to comply with all investigational requirements.

Following the assessment of eligibility for the study, individual consent was obtained from each subject’s parent/carer.

After the collection of data for the normative study, FLI-P data obtained previously from a small number of individual subjects with differing levels of hearing, drawn from The Shepherd Centre’s data, were plotted against the normative data. In all cases, individual consent for the use of these subjects’ data had been obtained.

#### 2.2.2. Sample Size

As the collected data were intended to form a normative data set, significant attention was paid to sample size calculation and data quality control.

The primary endpoint of this study was a successful collection of FLI-P outcomes for a minimum number of children within each 6-month age group from 0 to 6 years.

An analysis was conducted to determine the sample size of the FLI-P normative data set that would be needed to estimate the median FLI-P score as a function of age such that the error in the estimated median would be no greater than 2 (on the 0 to 64 scale) at every age 1, 2, …, 72 months with a probability of at least 90%.

The sample size calculation used Monte Carlo simulation, which involved generating a large number of random data sets based on certain assumptions, with the probability of the estimated medians having error no greater than 2 at all ages (from 1 to 72 months) estimated by the proportion of data sets in which that occurred. The sample size of the simulated data sets was varied to find the sample size required for a probability of 90%.

The simulation also involved two parameters, each controlling for a different source of variability in the scores: one being variation between children in the rate of increase of score with age, and the second being the variation (unreliability) within children. The two parameters then control the amount of variation between children in the rate of increase of score with age, whilst the other parameter controls the amount of variability in the child’s score at a particular age.

The simulation suggested that for the estimation of the median, a sample size of 144 children would be sufficient. While the sample size calculation was based on the estimation of the median, the estimation of the 16th and 84th percentiles was also checked, and the sample size of 144 also had a high probability (over 80%) of estimation of those percentiles with error no greater than 2 at each age. However, it was noted that a larger sample size would be required to protect against the possible effects of inaccurate assumptions in the simulation method and to address the issue of a possible high withdrawal rate. In addition, other percentiles (5th, 10th, 90th, and 95th) were to be estimated, and more extreme percentiles require a larger sample size.

It was anticipated that an enrolment rate of 50% of those cases approached could be achieved, considering the following factors:

- Proportion of parents who indicated an interest in participation;
- Proportion of children who meet eligibility criteria for investigation;
- Proportion of parents who successfully entered data into the Qualtrics website.

Data were collected into a Qualtrics database for children across the age range from birth to 6 years of age, split into 6-month age blocks (i.e., 12). Within each age block, the minimum number of children required per block was calculated to be *n* = 36 based on the statistical analysis and simulations. This resulted in a minimum total enrolment recruitment requirement across all ages of *n* = 432, with the proviso that a minimum of 36 subjects were required for each of the 12 separate age brackets.

Initially, up to 1500 families were identified from the MARCS Institute BabyLab database, and an invitation email was sent to each family to provide up to an anticipated 750 interested participants and, ultimately, the required number of data point entries into the Qualtrics database.

During the first few months of enrolment, it was noted that the overall response rate was relatively low. To address the low enrolment rate, eligibility failures, and the required minimum subject enrolment by age stratification for statistical analysis and significance across each age bracket distribution of numbers by age stratification, it was agreed that an expanded recruitment pool was needed to be invited to participate. Two additional investigational sites, the Macquarie University Child Language Lab and The Shepherd Centre, were added as recruitment sites. The Clinical Investigation Protocol was duly amended, and the additional investigative sites were approved by the respective Human Research Ethics Committee. This resulted in a significant increase in overall recruitment.

Subsequently, it was noted that due to the increased enrolment and data entry procedures on Qualtrics, in some of the age brackets, more than the required number of 36 subjects had been enrolled and data entered, whereas, in other age brackets, the minimum required number of children to be enrolled had not been achieved.

This was due to a decision not to close enrolment across the three investigative sites once the minimum number of subjects within each specific age bracket had been reached. Enrolment was continued until the minimum required number of subjects had been met for each of the 12 age blocks. As a result, a higher total number of subjects were recruited than the original minimum number originally approved. A retrospective ethics submission was made and approved by the respective HRECs to increase the sample size to 800 to accommodate the additional data points.

All data collected across the 12 age blocks were included in the statistical analysis. This meant that while the minimum target of 36 subject data entries for each age block was met, in some age blocks, a higher number of subjects was included, and the total number of subjects recruited into the study was increased.

#### 2.2.3. Bias Minimisation

There was no specific action taken to address bias, as this was a single-visit study for each subject. No comparator device was assessed as part of this clinical investigation.

#### 2.2.4. Data Quality Assurance

Data were collected and stored digitally. For each study participant, the parent’s/caregiver’s name, email, location, and study ID were collected. Background information on the child, including date of birth, confirmation of inclusion criteria and exclusion criteria, and ethnicity, was recorded and entered. Formal parental consent was recorded and entered for each subject.

Instructions and examples of data collection using the FLI-P were provided in written form for parents/caregivers. Instructions were also provided to guide the parents/caregivers in how to enter data into the FLI-P Study Qualtrics platform for parents/caregivers.

Data was entered directly into the FLI-P Study Qualtrics platform on a PC, tablet, or online device. Qualtrics is a secure online platform previously used by MARCS BabyLab to obtain digital consent for clinical investigations. Site personnel were also trained to use this system.

The investigation-specific data in the Qualtrics platform and FLI-P application could only be accessed by personnel allocated an account, which included site personnel, the Clinical Project Manager, Investigation Monitors, and Data Management personnel. Data validity was confirmed by the investigator through an electronic signature. An audit trail was kept by this system, and data clarifications were generated by the system and sponsor personnel after review of the data. Source data verification and source data review were conducted at the investigational site by the monitor to ensure the data quality.

The FLI-P application generates a unique identification code per subject, and only de-identified data were available to the sponsor, their representatives, and the investigators involved to protect patient data privacy.

Data entry and adherence to the Clinical Investigation Protocol were reviewed by an independent study monitor.

### 2.3. Statistical Methods and Analyses

As an assumption of normality was unlikely, quantile regression was used to estimate the 5th, 10th, 16th, 50th, 84th, 90th, and 95th percentile scores as functions of age. The 16th and 84th percentiles were included as in a normal distribution; they most closely approximate the mean minus and plus one standard deviation of the normal distribution.

The fitted functions were of the form:

$$FLI-P\;score=a_{1}+\frac{a_{2}-a_{1}}{1+\exp\left(-a_{3}\left(x-a_{4}\right)\right)’}$$

where *x* is the child’s age in months, and *a*_1_, *a*_2_, *a*_3_, and *a*_4_ are parameters to be estimated. The parameter a_2_ represents the upper limit of the function as age increases.

The statistical analysis was performed using the package quantreg, running under R to fit the quantile growth curve; ggplot2 (25) was used for plotting the trajectory of the range of percentiles [58,59,60].

### 2.4. Ethical Approval

This study was approved by and conducted under the oversight of the Western Sydney University Human Research Ethics Committee (WSU HREC), Approval Number H11517, dated 4 September 2017 (see Annexure 14.1). Informed consent for all subjects was obtained digitally using the approved Patient Informed Consent (PIC) on the Qualtrics platform.

## 3. Results

Data were collected for each child and allocated to the appropriate 6-month age bracket. For each child, the values for analyses were the age in months and FLI-P score entered. In total, *n* = 561 data points were entered. This number exceeded the original targeted total number of 432 required subject datasets. As noted previously, the increase in the total number of subjects recruited into the study was necessary to ensure the minimum recruitment requirement of 36 subjects in each of the 12 age brackets was met.

Additional information was also collected about the primary language spoken at home, any bilingual input, and the number of hours of exposure/week to a language other than English were also recorded. A range of second languages were reported, including Cantonese, Hindi, Spanish, Dutch, Tamil, Mandarin, Russian, Japanese, Arabic, Vietnamese, French, Croatian, Macedonian, Greek, Tagalog, Creole, Urdu, Assyrian, Tongan, Maltese, Yugoslav, Turkish, Czech, Gujarati, Swedish, Afrikaans, Hungarian, Bengali, TeoChew, Igbo, Hungarian, Italian, Maori, Bahasa, Italian, Serbian, Polish, Albanian, Armenian, Persian, Filipino, Ukrainian, German, Thai, Korean, Farsi, Punjabi, Swahili, Fijian, Burmese, Telugu, and Tongan. In most cases, exposure to a second non-English language was reported to be limited to less than 10 h per week.

Table 1 shows the target number of children intended for recruitment in each age bracket as originally estimated to enable appropriate statistical analysis and the actual number of children recruited into each of the 12 age brackets. As shown, the minimum requirement of 36 individual children was met for each of the 12 age brackets.

The youngest subject in the dataset was 1.5 months of age. It was noted that there were fewer data entries in the lowest age bracket. This was expected as completion of the SWISH program before enrolment was one of the inclusion criteria.

Higher numbers of children were recruited into age brackets from 7–12 months through to 49–54 months. Overall, there were a total of 536 child data points entered into the database across the 12 separate 6-month age brackets.

**Figure 1:**
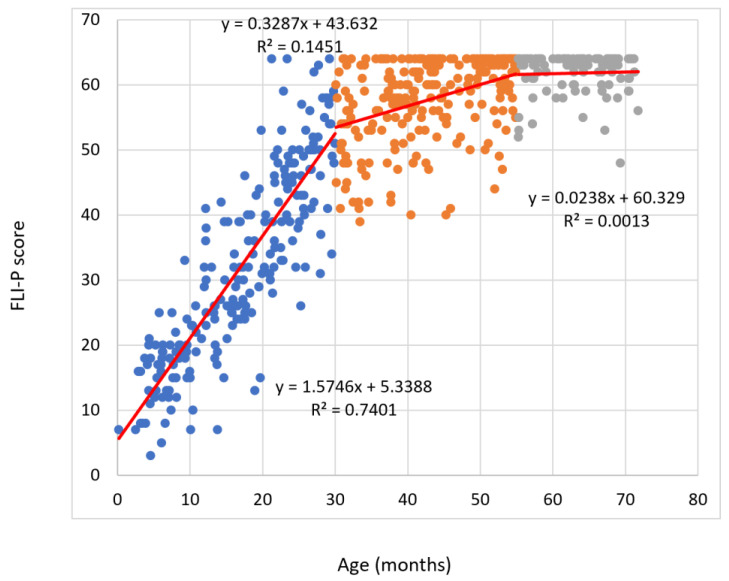
Data plot indicating age in months vs. totalFLI-P scores. A line of best fit was calculated for three distinct sections of the data, 0–30 months (blue), 31–54 months (orange), and 55–72 months (grey).

Figure 1 displays individual data points for each child. Whilst there was individual variability in the acquisition of skills, as would be expected, most skills were acquired before 30 months, or maximally 36 months, with subsequent higher-level skills mostly acquired by 54 months (4.5 years).

The trajectory of acquisition of skills for earlier chronological items was significantly steeper in contrast to later skills, reflecting the fast acquisition of auditory behaviours in the children’s first 30 months of life. The flattening of the trajectory for older typical-hearing children may be interpreted as consistent with the increased complexity of functional tasks and the use of information associated with the typical development with increasing age.

### Fitted Functions and Percentile Analysis

Statistical analysis was provided by an external consultant with expertise in the analysis of large databases. As noted, quantile regression was used to fit percentiles of FLI-P scores as functions of age. As the FLI-P scores were not normally distributed, the 5th, 10th, 16th, 50th, 84th, 90th, and 95th percentiles of the FLI-P scores were fitted as functions of age (Table 2).

The final estimated parameter values are shown in the following table.

**Figure 2:**
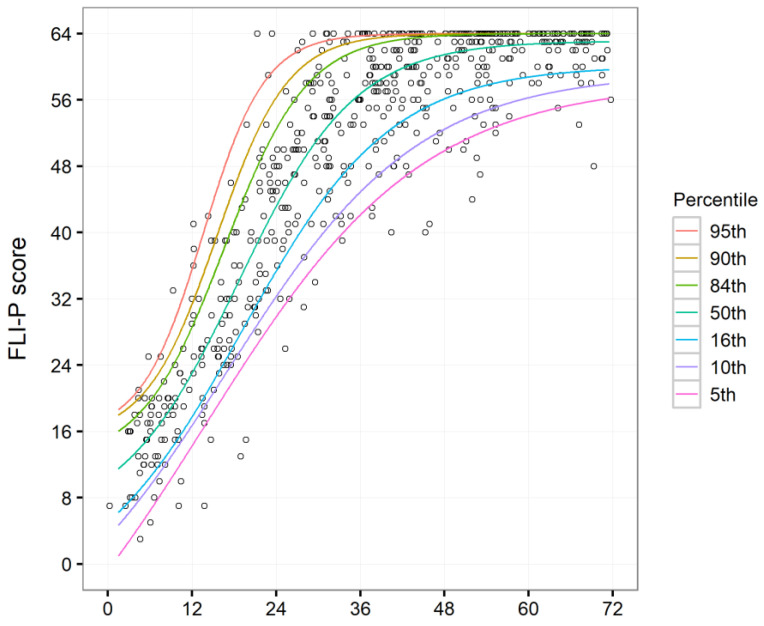
Individual scores by age (months). Calculated trajectory functions are shown for each percentile from the 5th through the 95th.

The individual data from Figure 1 and the fitted functions used to estimate the values of percentiles for any value of age up to 72 months are shown in Figure 2.

The youngest age entered into the data table was 1.5 months, and as evident in Figure 2, there were fewer children aged under 3 months as compared to older age groups. Given this observation, it was recommended that a starting point for the estimated percentiles based on the available normative dataset would be set at a minimum of 3 months rather than 1 month.

Figure 2 also shows the fitted functions for each percentile, together with the data points from the 561 children.

**Figure 3:**
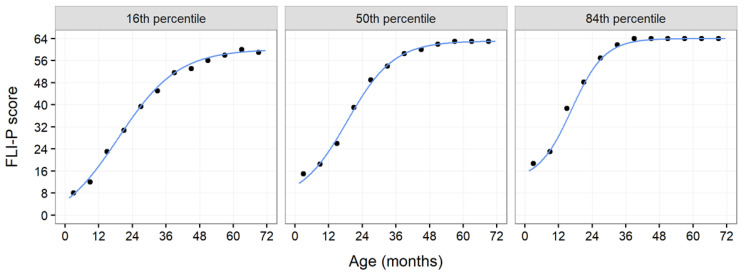
Sample percentile scores from 16th, 50th, and 84th percentiles and fitted functions.

To ensure the accuracy of the fitted functions, sample percentiles in the 6-month age groups (e.g., 0 < age < 6, 6 < age < 12, up to 66 < age < 72) were calculated. The plots in Figure 3 show these sample percentiles (dots) together with the fitted functions (lines). The sample percentiles were plotted at the midpoints of the age intervals (i.e., the sample percentile for 0 < age < 6 months bracket is plotted at age 3 months). This serves as a rough check, as the sample percentiles were calculated for the groups, while functions estimate the percentiles as continuous functions of age. There is strong agreement between the fitted functions and the sample percentile values in each of the age brackets across all percentiles assessed, as shown by the data for the 16th, 50th, and 84th percentiles.

A secondary objective of this study was to investigate how normative listening data can facilitate tracking individual progress and inform clinical recommendations and decision-making. To illustrate this practical application of the generated normative dataset, several examples are provided below.

**Figure 4:**
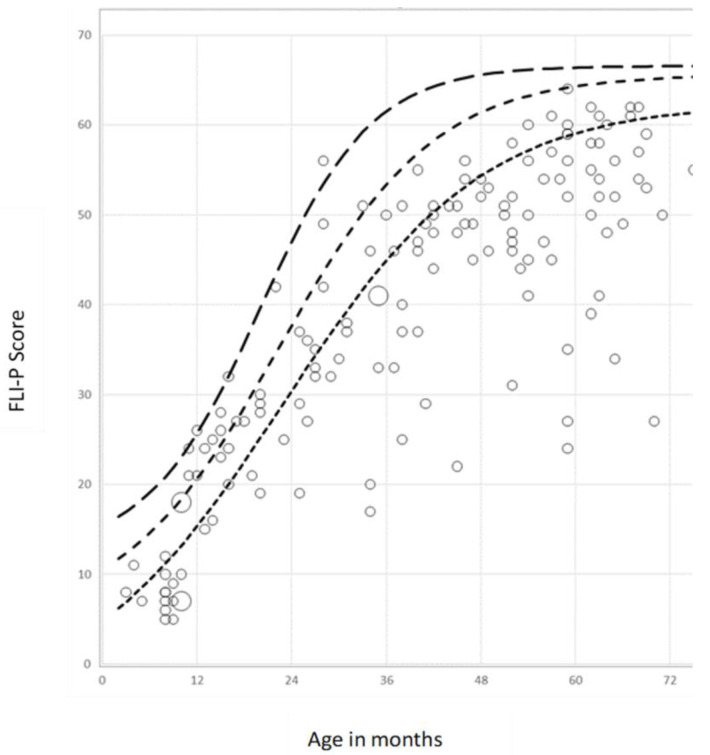
Data points(marked as circles) recorded by clinicians for children enrolled in an early intervention program for FLI-P score (*y*-axis) versus age at assessment in months (*x*-axis). The three dotted lines represent the 84th, 50th, and 16th percentile fitted functions for FLI-P scores against age as drawn from the normative dataset.

Figure 4 illustrates the application of the normative dataset in assisting the analysis of the current status of acquisition and development of listening skills for children with differing degrees of hearing loss enrolled in an early intervention centre.

In Figure 4, the fitted functions for the 84th, 50th, and 16th percentiles from the normative dataset are plotted as dotted lines (dotted lines). FLI-P scores for individual children, as recorded at specific ages by clinicians from an early intervention centre, are shown as individual points on the graph. For any individual child, functional listening skills, as measured by the FLI-P at that particular age, can be compared to those percentiles of FLI-P scores obtained from the normative dataset for the same age. The data also enable comparison of a cohort of subjects against the normative data, providing information that could be used to evaluate the acquisition of language skills for cohorts of children enrolled within an early intervention program.

Whilst the results in Figure 4 illustrate how a child’s current listening skills, as measured at any specific age, can be plotted against the normative data for typically-hearing children at the same age, the normative data also provide the potential for a child’s ongoing trajectory of the development of listening skills to be measured over multiple FLI-P assessments taken at sequential chronological age points. These data can then be compared and plotted against the projected trajectories for typically hearing children over the 0–72 months, and examples of this are presented in the Discussion.

## 4. Discussion

The primary endpoint of this study was the successful collection of FLI-P scores for a minimum number of typically hearing children within each 6-month age group from 0 to 6 years to enable the calculation of a normative dataset. Secondarily, results showed that parents/primary caregivers of individual children across all age brackets up to 6 years of age were able to complete the FLI-P for their child using written instructions and examples provided by the investigators without oversight or direct involvement of early intervention professionals.

The production of the normative dataset was intended to enable estimation of the median FLI-P score as a function of age, with an error no greater than 2, and to provide an accurate prediction of the 84th and 16th percentile scores for each age bracket. This data provides a set of FLI-P scores for typically hearing children across a range of ages from 3 months to 6 years of age. The generated data enabled a graphical representation of the normative dataset to be drawn, against which any individual child’s progress could be compared and assessed in comparison to typically hearing peers.

The FLI-P is potentially a more efficient and effective measure of listening skills compared with current clinical assessments of auditory skills, such as objective speech perception assessments, which cannot be reliably conducted in young children. It also provides a measure of developmental progress between birth and 6 years, which, at present, cannot be measured across this full age range with other clinical tools. FLI-P is also based on assessing functional listening—i.e., whether or not a child can make use of acoustic information through listening to enable them to perform specific functional activities—in contrast to specific checklists or measures of the acquisition of vocabulary items. In addition, due to its structure, the sensitivity of the FLI-P is comparatively less impacted by ceiling effects and less limited by the repetition of the same set of questions at different ages as observed for tests such as the PEACH [41] scale (to assess the effectiveness of young child’s hearing in the real world). In contrast, the FLI-P has a structured hierarchical range of listening skills expected to be acquired as the child’s auditory and listening skills develop chronologically.

As such, this provides a trajectory of the anticipated chronological sequence of listening skill development. While other subjective listening skill checklists have been developed, for example, the IT-MAIS [61] or LittlEARS [62], there is no single tool available that can be applied across the entire age range from birth to six years of age inclusively which covers the critical ages for language development.

Importantly, the FLI-P focuses on functional use and incorporates a cognitive assessment component, as it focuses on how the acquisition of listening skills impacts the child’s behaviour and learning ability rather than simply being an assessment of detection and/or imitation of sounds. Given that evidence shows that some 30% of children with hearing loss have comorbid disabilities [63,64], it is also important that an assessment tool can be used across a wide anticipated range of individual performance capabilities to holistically guide management decisions.

The FLI-P is sensitive to listening in children in early intervention with any level and configuration of hearing loss and across any type of hearing device. It has similarly been shown to be sensitive to children with and without comorbidities, enabling the tracking of their individual trajectories [47,48].

While it is important to measure a child’s progress at a given age against norms, it is equally relevant to plot the trajectory of an individual child’s acquisition of listening skills over time and compare these with expected growth based on normative data. This approach not only guides management but also provides a comprehensive view of the child’s development.

**Figure 5:**
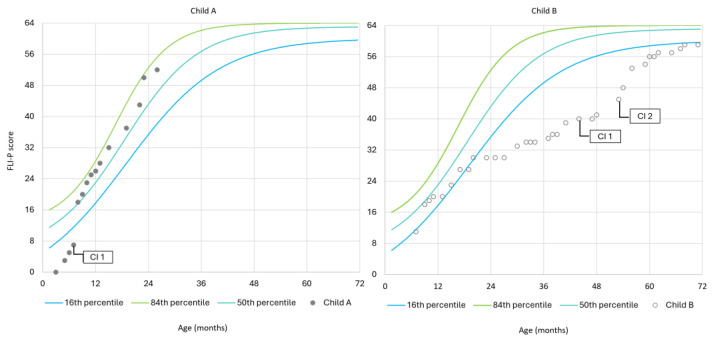
**Panel A**: Trajectory of scores over time, plotted against normative trajectories for 16th, 50th, and 84th percentile. The star represents the timing of the activation of the cochlear implant at 7 months. **Panel B**: Trajectory of scores over time, plotted against normative trajectories for 16th, 50th, and 84th percentile. The stars represent the timing of the activation of the sequentially implanted cochlear implants at 41 and 56 months.

The potential benefits of the normative data in tracking listening development over time are illustrated in Figure 5, which shows the FLI-P trajectories as measured at different intervals over time for two individual children. Both of these children were diagnosed with hearing loss at their newborn hearing screening and were subsequently enrolled in an early intervention centre. The FLI-P scores from individual assessments at different age points are plotted in Panels A and B, together with the 16th, 50th, and 84th percentile trajectories from the normative dataset.

In Panel A, the trajectory of individual scores is shown for Child A, who was diagnosed with bilateral profound sensorineural hearing loss at birth and enrolled in an integrated early intervention program. Child A received bilateral simultaneous cochlear implantation before six months of age, and immediately following implantation, the trajectory of scores rose rapidly to the 50th percentile of the normative data, following the trajectory up to 24 months of age. Comparing the FLI-P scores of Child A to the normative data indicates that the development of listening skills closely aligns with that of hearing peers at the same ages, demonstrating that early intervention and exposure to sound through bilateral cochlear implants support listening growth development.

In Panel B, the trajectory of individual FLI-P scores is shown for Child B, who was born with bilateral moderate–severe sensorineural hearing loss, fitted with hearing aids at 2 months of age, and enrolled in an integrated early intervention program before the age of six months. As shown, initially, Child B’s FLI-P trajectory was within the 16th to 84th percentile envelope of FLI-P scores from the normative data up to approximately 15 months of age. At this point, Child B’s FLI-P score trajectory diverges from the expected envelope of listening skill trajectories and plateaus approximately 2 SDs below the mean for a period of time. During this period, Child B experienced intermittent episodes of middle ear effusion. Following medical intervention with ventilation tubes, Child B’s FLI-P scores indicate resumed progress in the acquisition of their listening milestones; however, it is evident from the graphic that the rate of acquisition was slower and continued to diverge from the FLI-P score envelope as taken from the normative data. This was found to be consistent with outcomes in annual standardised speech and language assessments, and as a result, Child B was referred for cochlear implant evaluation and subsequently implanted with bilateral cochlear implants at 41 months and 56 months of age. Following implantation, Child B’s FLI-P score trajectory begins to converge with the normative data’s trajectory envelope. By the time Child B completes early intervention and is ready to start Kindergarten, their FLI-P score aligns with that of typically hearing peers. Figure 5 panel B highlights the sensitivity of FLI-P score trajectories in tracking individual progress, allowing for the early identification of a lack of progress and providing evidence-based information to parents for making informed decisions about their child’s intervention and monitoring subsequent progress.

These two case examples illustrate the potential application of FLI-P trajectory scores in providing valuable insights into an individual child’s language skill acquisition compared to the normative hearing population. This information is instrumental for parents and clinicians in assessing the efficacy of current early intervention strategies and making informed decisions about the child’s management.

The use of the FLI-P as a measure of the trajectory of listening skill acquisition over time against the normative data also ensures that individual variation in a parental or clinician assessment of a child at a particular age would have less impact than might be the case with a tool, which only gave a single assessment point for a child against norms.

### Limitations

It should be noted that the inclusion criteria for the typically hearing dataset included a Pass on a newborn hearing screening test, which would be primarily relevant to children in the younger cohorts of the subject group at the time of assessment. A second inclusion criterion was an absence of any parental or professional concern regarding hearing status. However, no specific diagnostic threshold hearing assessment was conducted for each child whose parents participated in providing FLI-P data.

In addition, while the number of subjects for whom FLI-P data was collected was sufficient to enable statistically valid percentile trajectory scores against age to be calculated, the collection of a larger pool of normative data from a broader population of children would increase the confidence in these normative percentiles. It is also noted that the percentile trajectories were for individual assessments of typically hearing children, as opposed to chronological FLI-P measures for individual typically hearing children taken over the entire age range from 0–72 months of age.

A further limitation is that the data were collected from different investigative sites, all located within a single major metropolitan centre in Australia, and the test items were written in English. As disclosed in the Methods section, a significant proportion of the parent group responding spoke more than one language. While it is relevant to note that the test items of the FLI-P are focused on the use of hearing for the development of language rather than the perception of specific language items per se and thus can be administered in any language, the effects of bilingual input to the language development of individual children were not assessed in this study.

Therefore, the normative dataset as collected may not be suitable for benchmarking FLI-P results for children from other countries with different language and cultural demographics. Expanding data collection to include diverse geographical and cultural populations would enhance the applicability and robustness of the normative data, and work is currently in progress to translate and modify the FLI-P for broader international application.

## 5. Conclusions

The data collected from a large population of typically hearing children provide a normative dataset of FLI-P scores across an age range of 0–6 years of age. Statistically valid data were collected, enabling percentile scores as a function of age to be calculated, and the 16th and 84th percentiles were chosen as the upper and lower trajectories to be used in comparisons against the normative dataset. The use of these trajectories enables comparison of the median and other selected percentile scores as a function of age, which can be presented as a graphical representation against which an individual child’s progress can be plotted and assessed. Trajectories for individual children with hearing loss, when compared to the normed data, were found to provide important information that could be useful in guiding early intervention decisions.

## Figures and Tables

**Table 1:** Number of participants (target and actual).

	0–6	7–12	13–18	19–24	25–30	31–36	37–42	43–48	49–54	55–60	61–66	67–72	Total
Target	36	36	36	36	36	36	36	36	36	36	36	36	432
Actural	36	40	42	49	42	47	63	46	53	36	36	36	536

**Table 2:** Estimated parameter value for percentile scores.

Percentile	*a_1_*	*a_2_*	*a_3_*	*a_4_*
5th	−25.3196	58.1656	0.0641	13.6060
10th	−13.2335	59.2565	0.0728	16.8049
16th	−4.2314	60.1019	0.0938	18.9745
50th	4.9005	63.1047	0.1204	18.5796
84th	12.2876	64.0000	0.1691	16.5906
90th	15.1011	64.0000	0.1978	15.5172
95th	15.7385	64.0000	0.2277	13.5759

**Figure A1:**
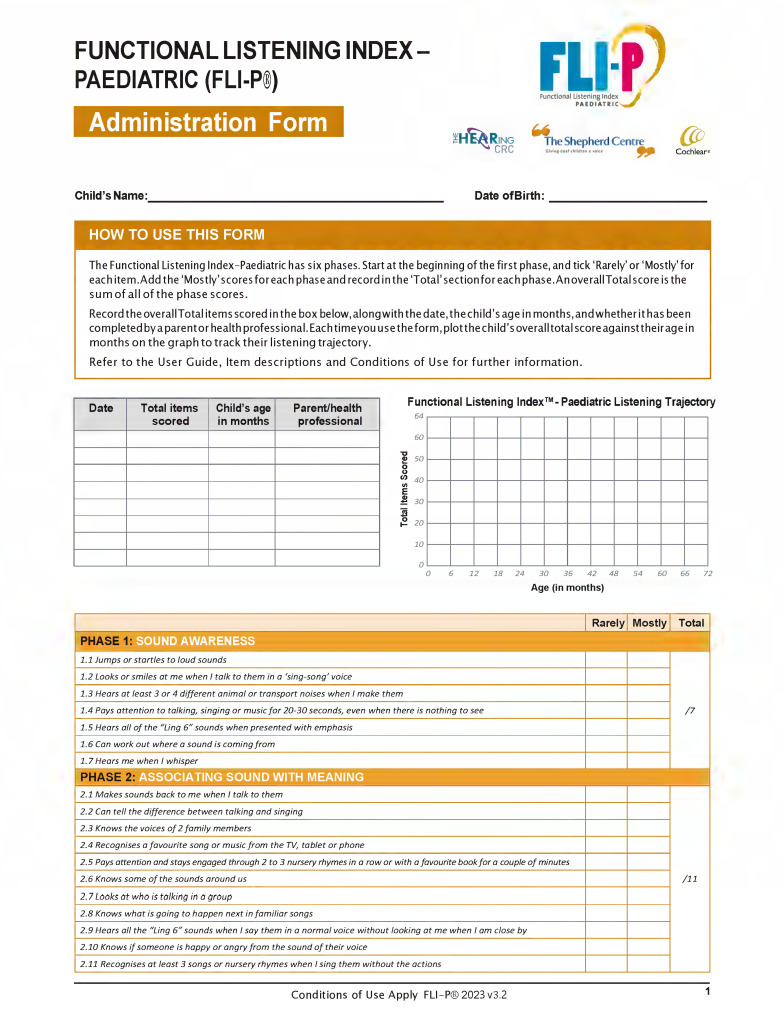

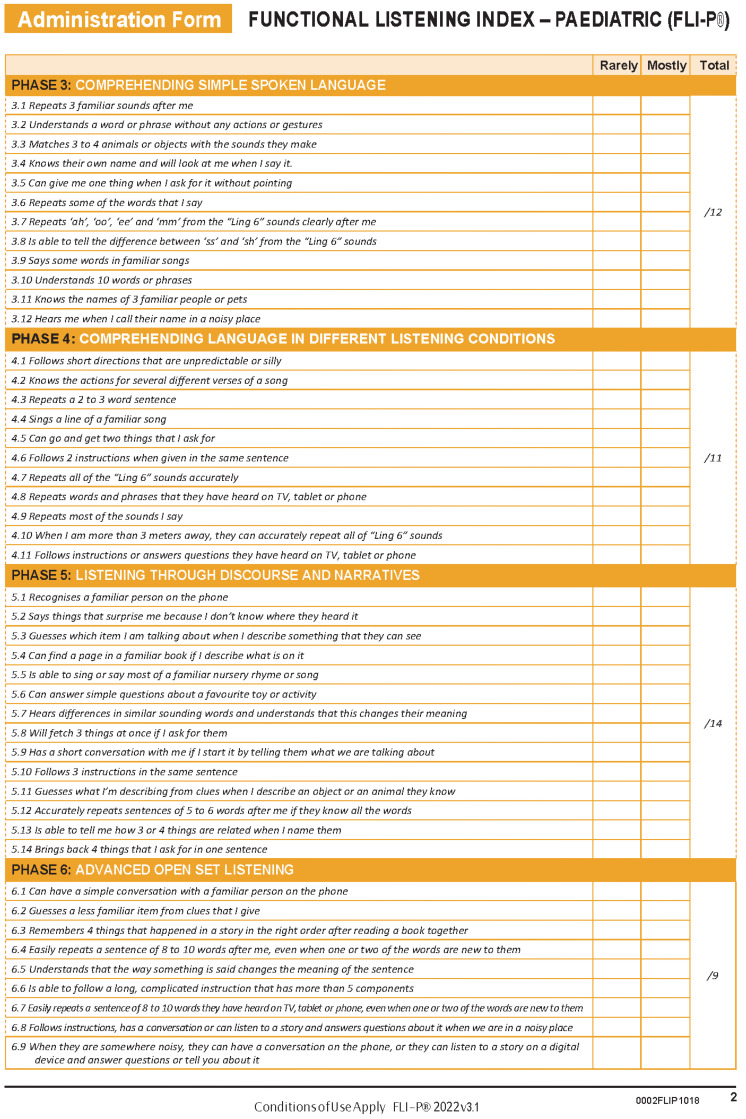
FLI-P administration form.

## Data Availability

The dataset comprising the FLI-P Normative Dataset is the subject of commercial arrangements which apply to its use and availability. The corresponding author will consider any request regarding the data supporting the conclusions of this article subject to the terms of that commercial arrangement.
